# Exploring the Expression of BCAS3 in Head and Neck Squamous Cell Carcinoma and Its Association With Prognosis

**DOI:** 10.7759/cureus.50995

**Published:** 2023-12-23

**Authors:** Devanand Challa, Chandra Pandi, Balachander Kannan, Vijayashree J Priyadharsini, Paramasivam Arumugam

**Affiliations:** 1 Oral Medicine, Saveetha Medical College and Hospital, Saveetha Institute of Medical and Technical Sciences, Saveetha University, Chennai, IND; 2 Centre for Cellular and Molecular Research, Saveetha Dental College and Hospitals, Saveetha Institute of Medical and Technical Sciences, Saveetha University, Chennai, IND

**Keywords:** diagnosis and prognosis, novel biomarker, cancer-genetics, oral cancers, oral health

## Abstract

Background

Head and neck squamous cell carcinoma (HNSCC) is a prominent global cancer that manifests across diverse sites such as the oral cavity, oropharynx, and larynx. Human papillomavirus (HPV) infection and genetic alterations contribute to HNSCC development.

Objective

To investigate the complex role of breast carcinoma amplified sequence (BCAS3) in HNSCC pathogenesis.

Methods

We used multiple databases to analyze BCAS3 expression in HNSCC using The Cancer Genome Atlas-Head-Neck Squamous Cell Carcinoma (TCGA-HNSC) dataset and validated it in oral squamous cell carcinoma (OSCC) using reverse transcription-quantitative polymerase chain reaction (RT-qPCR). The BCAS3 gene and protein networks were analyzed to identify their functional pathways.

Results

The results revealed significant overexpression of BCAS3 was observed in HNSCC and OSCC tumors. Our study explores BCAS3’s correlation with clinicopathological features and patient prognosis, suggesting its involvement in tumor aggressiveness. Notably, BCAS3 expression in HPV-positive and HPV-negative HNSCC samples emphasizes the intricate viral interactions. Kaplan-Meier plots demonstrate BCAS3’s impact on patient survival. Furthermore, BCAS3’s association between tumor immune infiltration and autophagy was uncovered.

Conclusion

Our study contributes to the understanding of BCAS3’s role in HNSCC and suggests its potential as a therapeutic target and diagnostic marker for these malignancies.

## Introduction

Globally, head and neck squamous cell carcinoma (HNSCC) is among the most prominent cancers. As of 2020, GLOBOCAN data are among the most prevalent human cancers in South Asian countries [1]. HNSCC can manifest in various sites, including the oral cavity, oropharynx, nasopharynx, hypopharynx, and larynx. Oral squamous cell carcinoma (OSCC) is a major subtype of HNSCC. Notably, tobacco and alcohol consumption are major contributing factors to HNSCC development [2]. In oropharynx tumors and certain other HNSCC cases, human papillomavirus (HPV) has been detected in approximately 25% of patients, while two-thirds of the patients diagnosed have advanced stages [3,4]. In addition to tobacco and alcohol consumption, certain sexual behaviors and non-smoking and non-drinking profiles have also emerged as risk factors for HPV-associated HNSCC [5,6]. An additional hallmark of HPV-related HNSCC is the elevated expression of p16 protein, with some studies suggesting its immunohistochemical expression as a surrogate marker for predicting treatment response [7,8]. Moreover, the development and progression of tumors are significantly influenced by genetic and epigenetic alterations [9,10]. The functional importance of non-coding regions in the genome for HNSCC development is well acknowledged [11,12].

The breast carcinoma amplified sequence (BCAS3), initially identified as amplified and overexpressed in breast cancer, shares 98% identity with murine Rudhira [13,14]. BCAS3 expression is driven by estrogen-positive breast cancer cells. Genetically, BCAS3 is fused with BCAS4 and is located on human chromosome 17q23, a region harboring several oncogenes with approximately 20% amplification in primary breast carcinomas. Similarly, earlier research indicated the presence of BCAS3 during angiogenesis and in human embryonic stem (ES) cells, suggesting its involvement in tumor angiogenesis [15,16]. Notably, BCAS3 overexpression has been correlated with tumor development [16]. BCAS3 is expressed in tumor-derived cell lines such as HeLa and MCF-7, is amplified in 9% of initial breast tumors, and is linked to breast cancer progression. Multiple oncogenes are located in the chromosomal region where BCAS3 resides, which is a target of metastasis-associated protein 1 (MTA1) [17-19]. MTA1 binds to the estrogen response element enhancer located 12 kbp downstream of the BCAS3 transcription start point. High BCAS3 expression and ER/PR positivity hinder tamoxifen response in breast cancer patients [20]. The BCAS3 gene stands out as a promising biomarker for breast cancer. However, the prognosis for HNSCC is currently quite unfavorable. It is crucial to identify new biomarkers for HNSCC, as there is currently no research on the involvement of the BCAS3 gene in this type of cancer. Consequently, our focus is on unraveling the role of the BCAS3 gene in HNSCC to address this gap in knowledge.

The main objective of this study is to identify the expression of BCAS3 gene in HNSCC. We analyzed the BCAS3 expression using The Cancer Genome Atlas-Head-Neck Squamous Cell Carcinoma (TCGA-HNSC) dataset and OSCC through the University of Alabama at Birmingham CANcer data analysis portal (UALCAN) database and RT-qPCR. Moreover, the BCAS3-involved pathways and functions were analyzed using its network through online tools. We employed The Cancer Genome Atlas (TCGA)-based UALCAN database for analysis of the gene expression and clinicopathological features of HNSCC and RT-qPCR analysis of the gene expression between BCAS3 and OSCC and protein network of the gene is analyzed by insilico tools.

## Materials and methods

Gene expression analysis using the UALCAN database

We utilized TCGA dataset to assess the expression of BCAS3. This investigation specifically concentrated on scrutinizing BCAS3 expression in HNSCC (n=520) and normal tissues (n=44) through The UALCAN database (http://ualcan.path.uab.edu) [21]. The clinicopathological features of HNSCC and the examination of BCAS3 expression were analyzed using the publicly available TCGA dataset.

Patient and sample collection

The study was conducted at Saveetha Dental College and Hospitals, Chennai, India from the period of February 2023 to August 2023. We obtained a total of 30 samples from individuals diagnosed with OSCC, a subtype of HNSCC. None of the patients had a history of systemic or genetic diseases, nor had they experienced cancer recurrence. Surgical procedures were employed to collect tumor and non-tumor samples, which underwent thorough validation by a pathologist through histopathological analysis. The collected samples were then stored at -80°C for subsequent analysis. Approval for this study was obtained from the Institutional Human Ethical Committee at Saveetha Dental College and Hospitals (Approval No: IHEC/SDC/FACULTY/20/PERIO/01), adhering to the principles outlined in the Declaration of Helsinki. Informed consent was obtained from either the patient or their guardian. Detailed clinical information about the patients is provided in Table 1.

**Table 1:** Clinical features of patients with oral squamous cell carcinoma RMT: Retromolar trigone; GBS: Gingivobuccal

S.No.	Variable	Category	No. of patients (%)
1	Gender	Male	24 (80)
		Female	6 (20)
2	Age	<50 years	13 (43)
		>51 years	17 (57)
3	Grade	Well-differentiated	17 (57)
		Moderately differentiated	12 (40)
		Poorly differentiated	1 (3)
4	Site	Buccal	9 (30)
		Tongue	7 (23)
		Other (RMT, GBS, Maxilla, Mandible)	14 (47)
5	Stage	Ⅰ	4 (13)
		Ⅱ	6 (20)
		Ⅲ	6 (20)
		Ⅳ	14 (47)
6	Laterality	Left	11 (37)
		Right	19 (63)
7	Pattern of invasion	Cohesive	17 (57)
		Non-cohesive	13 (43)
8	Invasion of adjacent sites	Present	12 (40)
		Absent	18 (60)

RNA extraction and complementary DNA synthesis

The extraction of RNA from both tumor and non-tumor tissues was carried out utilizing TRIzol reagent (Thermo Fisher Scientific, Waltham, United States). Assessment of RNA quality and quantity was conducted using Nanodrop One (Thermo Fisher Scientific, Waltham, United States). Subsequent to quantification, 2000 ng of total RNA was transformed into cDNA using the Takara 1st Strand cDNA Synthesis Kit (Takara, Tokyo, Japan) in accordance with the manufacturer's instructions.

Real-time quantitative polymerase chain reaction analysis

Real-time quantitative polymerase chain reaction (RT-qPCR) was employed to analyze gene expression utilizing the previously generated complementary DNA (cDNA). Primers, synthesized by Eurofins (Eurofins Scientific, Bangalore, India) based on literature [19], are detailed in Table 2. A PCR reaction volume of 25 µL was prepared, comprising 12.5 µl of 2x SYBR, 50 µM forward and reverse primers, 2 µL (200 ng) of cDNA template, and the remaining volume filled with DDH2O. All experiments were conducted using Bio-Rad CFX Opus 96 (Bio-Rad Laboratories, Inc., Hercules, United States). The protocol involved an initial denaturation at 95°C for three minutes, followed by 40 cycles of denaturation at 95°C for 10 seconds and annealing at 58°C for 30 seconds. GAPDH served as the reference gene. The Bio-Rad CFX Maestro 1.0 software (version 4.0.2325.0418, Bio-Rad Laboratories, Inc., Hercules, United States) automatically calculated the gene expression fold change. The primers used in this study are provided in Table 2.

**Table 2:** Primer sequence for RT-qPCR used in this study RT-qPCR: Reverse transcription-quantitative polymerase chain reaction

Gene	Forward primer	Reverse primer
BCAS3	5’- GAAGAATGGCTTTCCCAGGT-3’	5’- GTCACGCTCCTGTCAAAGG-3’
GAPDH	5’-TCCAAAATCAAGTGGGGCGA-3’	5’-TGATGACCCTTTTGGCTCCC-3’

Survival analysis via Kaplan-Meier plotter

The Kaplan-Meier plotter (https://kmplot.com/) [22] was utilized to examine patient survival data, integrating both gene expression and patient survival information. Specifically, in our study, Kaplan-Meier analysis was employed to explore the mRNA-level prognostic implications of BCAS3 in HNSCC.

Functional enrichment and network analysis using Metascape

Tumor IMmune Estimation Resource (TIMER2.0) (http://timer.cistrome.org) [23] was employed to assess the correlation between BCAS3 expression and tumor immune cell infiltration. For insights into gene functions, list analysis, and prioritization in functional assays, GeneMANIA (http://genemania.org) [24] was utilized. In our study, GeneMANIA was instrumental in examining the functional characteristics of BCAS3. The Search Tool for the Retrieval of Interacting Genes/Proteins (STRING) database (https://string-db.org/) [25], a valuable resource for information on protein-protein interactions, was incorporated to explore interactions involving BCAS3. Metascape (https://metascape.org/) [26], a robust bioinformatics tool, facilitated functional enrichment analysis, gene annotation, and network visualization. In our study, Metascape was employed to analyze data collected from GeneMANIA and STRING for BCAS3, leveraging multiple open-source resources and databases for comprehensive gene function analysis, biological process investigation, and pathway exploration.

Statistical analysis

IBM SPSS Statistics for Windows, Version 25 (Released 2017; IBM Corp., Armonk, New York, United States) was used for statistical analysis using the Student’s t-test or one-way analysis of variance. Statistical significance was set at p<0.05.

## Results

BCAS3 overexpression in HNSCC and OSCC tumors

**Figure 1:**
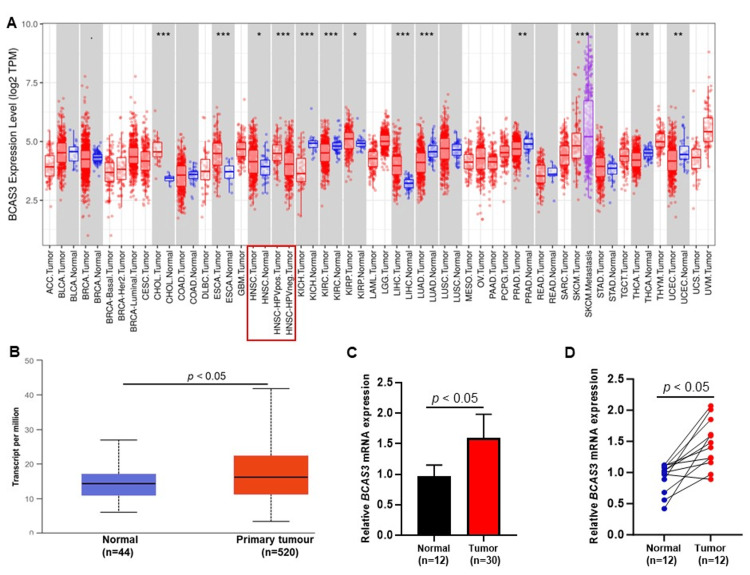
(A) Expression level of BCAS3 mRNA in various cancers (blue - normal, red - tumor). (B) The boxplot represents the expression pattern of BCAS3 between HNSCC tumors and normal tissues (blue - normal, red - HNSCC tumor). (C) The bar graph indicates that the OSCC tumor (red) had significant BCAS3 overexpression, and normal tissue (black) was revealed by RT-qPCR. (D) The plot represents BCAS3 expression in the tumor and matched normal tissue in the same patient, which also showed a significant difference in BCAS3 expression The x-axis represents sample type and the y-axis indicates levels of BCAS3 expression (A-D). TPM (transcripts per million) (A, B) and the fold change (C, D) were used to measure BCAS3 mRNA expression levels. Significance denotes *p<0.05, **p<0.01, ***p<0.001. HNSCC: Head and neck squamous cell carcinoma; OSCC: Oral squamous cell carcinoma; RT-qPCR: Reverse transcription-quantitative polymerase chain reaction

In this study, our initial analysis involved assessing BCAS3 expression across various cancers using online databases drawing data from the TCGA dataset. BCAS3 displayed distinct expression patterns across different cancer types, with a notable emphasis on HNSCC (p<0.05) (Figure 1A). Further exploration using the UALCAN database revealed a significant overexpression of BCAS3 in primary HNSCC tumors compared to normal tissues (p<0.05) (Figure 1B). Our investigation extended to validate BCAS3 expression in OSCC samples (n=30), a prominent subtype of HNSCC. We observed a substantial increase in BCAS3 expression in OSCC tumors (n=30) compared to non-tumor tissues (n=12, p<0.05) (Figure 1C). Furthermore, a noteworthy difference in BCAS3 expression within the tumors was evident when comparing the matched control samples (n=12, p<0.05) (Figure 1D).

Association of BCAS3 expression with clinicopathological features and HNSCC prognosis

**Figure 2:**
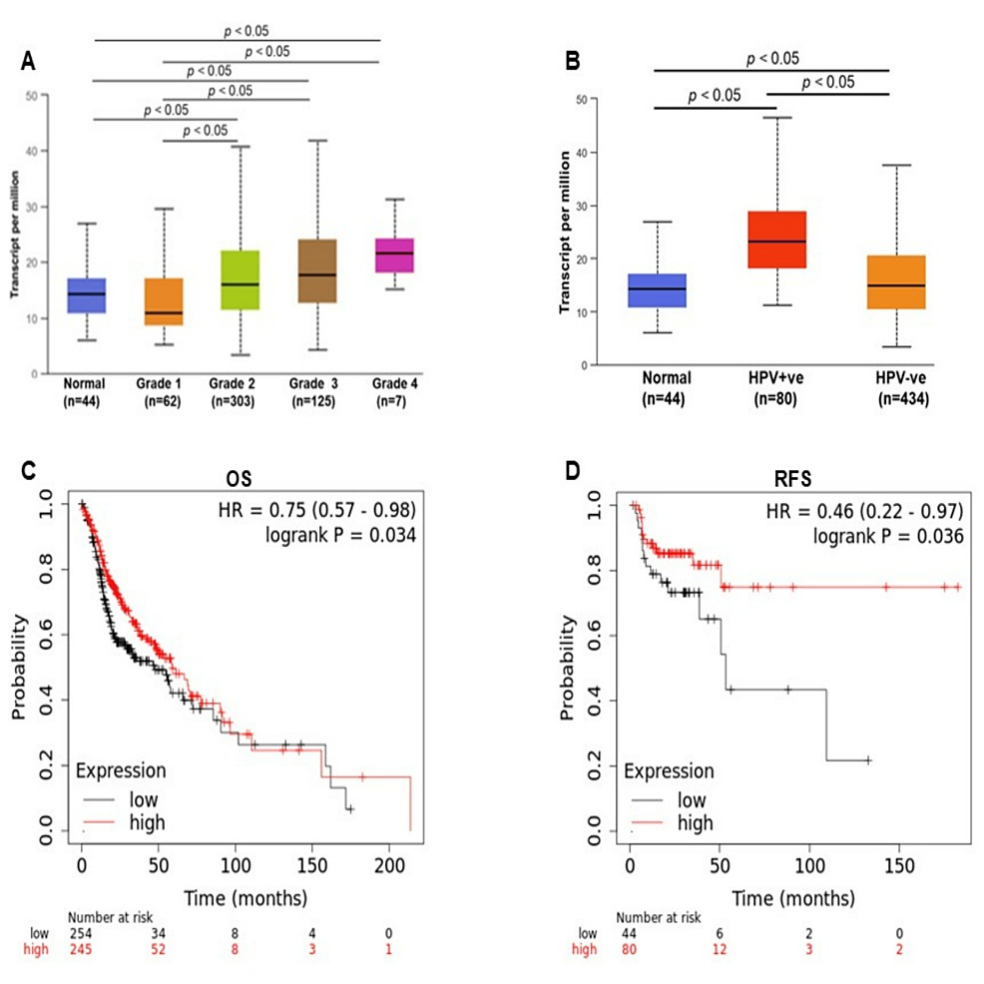
BCAS3 expression in HNSCC clinicopathological features. (A) The box plot represents the expression of BCAS3 in normal and different types of tumor grades in HNSCC. (B) Box plot representing BCAS3 expression in the HPV-based (human papillomavirus) HNSCC and normal samples. The x-axis represents the sample type, and the y-axis indicates the levels of expression. The Kaplan-Meier plot suggests that BCAS3 expression is significantly associated with (C) Overall survival (OS) and (D) Relapse-free survival (RFS); the red line indicates high BCAS3 expression, and the black line indicates low BCAS3 expression. The x-axis represents the month and the y-axis represents the probability OS: Overall survival; RFS: Relapse-free survival; HR: Hazard ratio; HNSCC: Head and neck squamous cell carcinoma; HPV: Human papillomavirus

Our study delved into the correlation between BCAS3 expression and the clinicopathological features and prognosis of patients with HNSCC. Notably, BCAS3 expression displayed a progressive increase in tandem with higher tumor grades (p<0.05) (Figure 2A). Additionally, a comparison of HPV-positive and HPV-negative HNSCC samples, alongside normal samples, indicated significantly higher BCAS3 expression in HPV-positive HNSCC (p<0.05) (Figure 2B). Utilizing Kaplan-Meier plots based on the HNSC TCGA dataset, a significant association between BCAS3 expression and both overall survival (OS) and relapse-free survival in HNSCC was identified (p<0.05) (Figures 2C, 2D). Furthermore, the impact of BCAS3 gene expression levels concerning tumor grade, stage, gender, and race in the context of HNSCC patient survival was evaluated. However, the results showed insignificance in these associations.

BCAS3 links to tumor immune infiltration and autophagy

**Figure 3:**
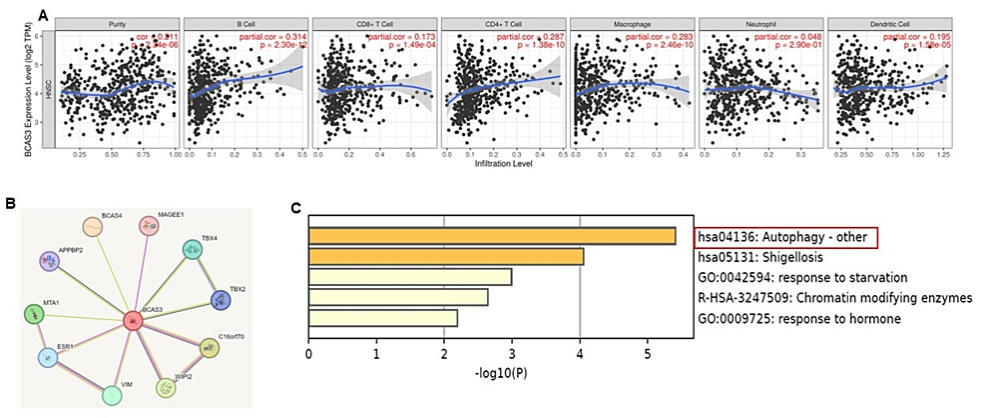
Tumor immune infiltration and functional role of BCAS3 network. (A) Correlation between BCAS3 expression in various immune cells of HNSCC, including B cells, CD8+ cells, CD4+ cells, macrophages, neutrophils, and dendritic cells. The blue line indicates whether it is positively or negatively correlated with the p-value. (B) Interaction network of BCAS3 protein. (C) Functional pathways of BCAS3 and its associated protein networks HNSCC: Head and neck squamous cell carcinoma; TPM: Transcripts per million

We extended our investigation to examine the relationship between BCAS3 expression and tumor immune infiltration, leveraging TIMER 2.0. The findings revealed a positive correlation between BCAS3 expression and B cells, CD4+ cells, and dendritic cells, while a negative correlation was observed with CD8+ cells and macrophages in the tumor immune cell infiltration of HNSCC patients (p<0.05) (Figure 3A). Furthermore, we identified a protein network associated with BCAS3, including BCAS4, MAGEE1, TBX4, TBX2, etc. (Figure 3B), for subsequent functional enrichment analysis. The analysis conducted using Metascape underscored the significant involvement of BCAS3 and its protein network in autophagy (Figure 3C).

## Discussion

HNSCC is a prominent global cancer with persistently low OS rates, underscoring the urgent need to discover novel biomarkers and therapeutic targets. Previously, we identified some novel genes that are associated with HNSCC progression and could be used as biomarkers and prognostic indicators [9,27]. In the present study, we explored the relationship between BCAS3 and HNSCC progression.

In particular, primary breast cancers demonstrate an amplification rate of 9.4% for the BCAS3 gene [13]. Intriguingly, BCAS3 overexpression has been found to impede the apoptosis process, thereby reducing the sensitivity of cancer cells to chemotherapeutic drugs such as paclitaxel and doxorubicin in MCF-7 cells. Notably, this overexpression of BCAS3 plays a role in the post-translational inactivation of p53 in breast cancer cells, a finding that has significant implications [20]. This collective evidence strongly suggests that BCAS3 overexpression not only promotes cancer cell proliferation but also fosters resistance to chemotherapy drugs, potentially complicating treatment outcomes.

Furthermore, a study conducted by Wang et al. illuminated the impact of BCAS3 across different cancer contexts. Their research revealed substantial overexpression of BCAS3 in glioblastoma multiforme (GBM), correlating with an unfavorable prognosis. This investigation encompassed both in vitro and in vivo models, revealing BCAS3 regulatory role in GBM cell proliferation and cell cycle progression, operating through the p53/GADD45α signaling pathway [28]. This finding provides a comprehensive perspective on BCAS3's potential as a key regulator across diverse cancer scenarios, underscoring its significance in the intricate landscape of cancer biology. Adding to this spectrum of effects, the study by Siva et al. underscored BCAS3's dynamic presence beyond malignancies. It was demonstrated that BCAS3 exhibits high expression levels in ES cells during their differentiation into blood vascular precursors. In brain tumors such as glioblastoma, hemangiopericytoma, and brain abscess, the observed elevated BCAS3 expression was not only within tumor cells but also the expression of the BCAS3 gene in vascular precursors derived from human ES cells has a role in the formation of tumor vessels [16]. This intriguing finding suggests that BCAS3 might play a pivotal role in the context of rapidly proliferating cells, potentially extending its influence to various cancerous environments.

Our study sheds light on BCAS3's multifaceted role in HNSCC. We observe significant BCAS3 overexpression in this tumor, aligning with its role in various cancers and indicating its significance in pathogenesis.

BCAS3's pronounced elevation in primary HNSCC tumors, validated in OSCC, suggests its potential as a distinctive prognostic and diagnostic biomarker for both malignancies. Our study's insights into BCAS3 expression correlations with clinicopathological features and patient prognosis highlight its possible involvement in tumor aggressiveness and complex interactions between viral factors and tumor development in HPV-positive and HPV-negative HNSCC. The association between BCAS3 expression and patient outcomes underscores its prognostic potential, requiring further mechanistic exploration. The link between BCAS3 expression and tumor immune infiltration is notable, indicating intricate interactions within the immune microenvironment. Functional enrichment analysis suggests BCAS3's role in autophagy processes, potentially impacting tumor growth and survival dynamics. Our study identifies Bcell, CD4+ cells, and dendritic cells are positively correlated with BCAS3 in HNSCC. CD8+ cells and macrophages are negatively correlated with BCAS3 in HNSCC. These cells are very crucial for HNSCC development, alternations in the immune cells severely impact with patient's prognosis and also affect the immunotherapy treatment [29]. HNSCC etiological factors such as cigarette smoke, tobacco, alcohol, HPV-16, and radiation exert carcinogenicity associated with autophagy [30]. Our results suggested that BCAS3 in HNSCC is related to autophagy but the molecular mechanism of BCAS3 in HNSCC related to autophagy is still unclear, so functional studies are required.

These outcomes align with prior research identifying BCAS3 as a prognostically significant biomarker in diverse malignancies such as breast and glioblastoma. Nevertheless, the precise mechanisms that drive BCAS3 involvement in cancer growth and dissemination remain elusive. Both the short sample size and the insilico analysis are limitations of this study. We need a lot of samples to do more in-depth research, such as studies on the amount of protein expression, in vitro and in vivo investigations, which can analyze the molecular mechanism of the gene. These investigations aid in understanding the BCAS3 gene's molecular mechanism in HNSCC. For future directions, it is advisable to expand this study by incorporating in-vivo conditions. Additionally, enhancing the comprehensiveness of the research would involve conducting functional studies specifically focused on autophagy and cancer signaling pathways.

## Conclusions

Our study elucidates the pivotal role of BCAS3 in HNSCC by highlighting its overexpression in this tumor. We have revealed its associations with clinicopathological features, patient prognosis, tumor immune infiltration, and autophagy processes. While these findings offer promising avenues for further research, the underlying molecular mechanisms governing BCAS3 effects on tumor progression remain an intriguing topic for future exploration. Ultimately, unraveling the precise role of BCAS3 could pave the way for novel therapeutic strategies and diagnostic approaches for HNSCC patients.
